# Public health digitalization in Europe: *EUPHA vision, action and role in digital public health*

**DOI:** 10.1093/eurpub/ckz161

**Published:** 2019-11-18

**Authors:** Anna Odone, Stefan Buttigieg, Walter Ricciardi, Natasha Azzopardi-Muscat, Anthony Staines

**Affiliations:** 1 School of Public Health, Faculty of Medicine, University Vita-Salute San Raffaele, Milan, Italy; 2 European Public Health Association, Utrecht, The Netherlands; 3 Digital Health Malta, Villetta, Malta; 4 Sezione di Igiene, Istituto di Sanità Pubblica, Università Cattolica del Sacro Cuore, Rome, Italy; 5 Department of Health Services Management, Faculty of Health Science, University of Malta, Villetta, Malta; 6 School of Nursing and Human Sciences, Dublin City University, Dublin 9, Ireland

## Abstract

**Background:**

As digitalization is progressively permeating all aspects of society, how can be it fruitfully employed to sustain the public health goals of quality, accessibility, efficiency and equity in health care and prevention? In this paper, we reflect on the potential of applying digital tools to public health and discuss some key challenges.

**Methods:**

We first define ‘digitalization’ in its broader sense, as well as applied to public health. We then build a conceptual framework where key public health domains are associated to available digital technologies in a matrix that help to identify digital features that bolster public health action. We also provide illustrative data and evidence on the application of digital solutions on selected public health areas. In the second part, we identify the strategic pillars for a successful European strategy for public health digitalization and we outline how the approach being pursued by the European Public Health Association (EUPHA) applies to digital health.

**Results:**

From a public health perspective, digitalization is being touted as providing several potential benefits and advantages, including support for the transition from cure to prevention, helping to put people and patients at the center of care delivery, supporting patient empowerment and making healthcare system more efficient, safer and cheaper. These benefits are enabled through the following features of digital technologies: (i) Personalization and precision; (ii) Automation; (iii) Prediction; (iv) Data analytics and (v) Interaction.

**Conclusion:**

A successful European strategy for public health digitalization should integrate the following pillars: political commitment, normative frameworks, technical infrastructure, targeted economic investments, education, research, monitoring and evaluation. EUPHA acknowledges digitalization is an asset *for* public health and is working both to promote the culture of “public health digitalization”, as well as to enable its planning, implementation and evaluation at the research, practice and policy level.

## Introduction

Early this year, the Topol Review, an independent study commissioned by the UK Department of Health and Social Care, provided a number of recommendations to ‘*prepare the healthcare workforce to deliver the digital future*’.^1^ The document outlines how technological innovation is likely to change medical practice, clinical staff functions and role, and how health services delivery and clinical staff training should be re-structured to best embrace digital innovation. Depending on our background, sensitivity and knowledge, for some of us ‘digital health’ is about technology; for others, it refers to new ways of communication or to automation in care. Although all these definitions are not necessarily wrong, it is important to pursue alignment and common vision about the added value of applying digitalization to health. ‘Digital’ refers to data or signals recorded, stored, expressed and transmitted as series of the digits 1 and 0 (signal present or absent). The shift from mechanical and analog technology to digital technology initiated the Digital Revolution which started during the so called 3rd Industrial Revolution and has continued during the ongoing 4th Industrial Revolution where digital technologies, such as artificial intelligence, genome editing, augmented reality, robotics and 3-D printing are rapidly shaping all aspects of society and, most importantly, ‘*the way humans create, exchange, and distribute value*’.^2^ This has impacts on health. There are two different dimensions to consider: the way digital tools can support and improve healthcare delivery; and the impact of societal digitalization on physical and mental health.^3^ With regard to the first dimension, there is no doubt that digital tools application to the different fields of medicine will dramatically improve diagnostics, treatments and ultimately health outcomes. When it comes to public health the process is less straight forward. In this paper, we aim at reflecting on the potential and challenges of applying digital tools to public health^4^, starting with one major premise: while clinical care is limited to diagnosis, cure and rehabilitation, public health encompasses all aspects of society, the environment, human and animal life. Public health stands largely outside hospitals and healthcare facilities; public health deals with health policy and management, with health education, health promotion and heath communication, with the physical and social environment, with animal health and food safety; public health adds prevention to the care paths. Similarly, as commented in a *Nature* editorial on the digital revolution ‘*An explosion in information technology is remaking the world, leaving few aspects of society untouched*’.^5^ Considering the broad spectrum covered by both public health and digitalization gives an idea of the additional layer of complexity when envisaging a framework for ‘public health digitalization’.

## Objectives and methods

The current paper first aims at defining ‘digitalization’ in its general sense, as well as when applied to health and public health. We highlight the added value of digitalization for public health, reinforcing that digitalization is not an aim in itself but a means to implement better public health programmes and policies. We then build a conceptual framework where key public health domains are associated to available digital technologies in a matrix that help to identify digital features that bolster public health action. Based on literature review data and consultations with experts, we provide illustrative data and evidence on the application of digital solutions on selected public health domains. In the second part of the paper we identify the strategic pillars for a successful European strategy for public health digitalization, narrowing down aspects related to: European and national-level political commitment, normative frameworks, technical infrastructure, targeted economic investments, training and education, research, monitoring and evaluation. We illustrate how the European Public Health Association (EUPHA) and its new Digital Health section can be a front runner in leading the change towards the digitalization of public health in Europe, also through the framework of EUPHA’s strategy.

## The added value of digitalization for public health

### Digitalization as a means, not an end in itself

Digitalization should not modify public health principles; rather it should support and enable their implementation. We see digitalization as a means, a set of tools, not an *aim* for public health. Digital public health is therefore not a discipline *per se* but an asset our community has to fulfill its aims and mission. The health system goals of quality, accessibility, efficiency and equity of healthcare, embraced by public health professionals are unaltered by the process of digitalization.^6^ Acknowledging that digitalization is a technological revolution permeating all aspects of society, how can it be fruitfully employed to sustain the ‘*science and art of preventing disease, prolonging life and promoting health and well-being through the organized efforts and informed choices of society, organizations, public and private, communities and individuals*’?^7^ As public health in Europe faces substantial challenges^8^—including the aging population, the burgeoning burden of non-communicable diseases, the influence of vested interests on their behavioral risk factors, the sustainability of national health services, and the health divide between and within countries, we should seek to better understand the potential of digital technologies in supporting the public health effort.

### Digital features which bolster public health action

The Topol review categorizes digital healthcare technologies as: (i) genomics, (ii) digital medicine, (iii) artificial intelligence and (iv) robotics (their detailed list and definitions are available as Supplementary appendix S1).^1^ Classification of digital technologies for public health is hampered by the rapid pace at which they develop, and by the paucity of comprehensive data on their application, adoption and impact. We propose a conceptual framework (table 1) that aims at summarizing the features and characteristics of digital technologies that can fruitfully be applied to the wide spectrum of European public health action; at all levels and in its different domains. In table 1, we summarize public health domains and, separately, we list the categories of available digital technologies that can be applied to them. This matrix exercise helps to identify and depict the digital solutions’ features that bolster and potentially add value to public health practice. These are: (i) Personalization and precision; (ii) Automation; (iii) Prediction; (iv) Data analytics (including big data and interoperability) and (v) Interaction. For these features, we provide selected examples and supporting evidence.

**Table 1:** The potential added value of digitalization for public health: a conceptual framework

Public health Pillars	Public health Domains	Digital Health Technologies^a^	Features	Potential Public health Benefits and advantages
Practice Research Training and Education Policy	Health prevention Health communication Health education Health promotion Health services organization, management and delivery Epidemiology and control of communicable diseases Risk management, hospital hygiene and safety Epidemiology and control of non-communicable diseases Food safety Environmental health Surveillance analysis and reporting Impact assessment monitoring and evaluation	Genomics Telehealth Smartphone apps (MHEALTH) Social media Wearables and sensors Virtual and augmented reality Drones Internet of things Big data Artificial intelligence (incl. predictive analytics, speech recognition and natural language processing) Robotics Distributed ledger technologies	Personalization and Precision Automation Prediction Data analytics (incl. Big Data, data transfer and interoperability) Interaction	Shift from cure to prevention Care closer to people People-centered care Safer, faster and more efficient services Less expensive care

Note: ^a^Refer to Supplementary appendix S1 for definitions.

#### 

##### Personalization and precision:

Although the concept of ‘Precision Public Health’ has been criticized,^9^^,^^10^ it is a fact that advances in data analytics and genomics allow us to *target* more effectively and efficiently public health interventions.^10^ It is increasingly recognized that several genome-based applications have the potential for tailored primary and secondary prevention.^11–13^ Also, training of healthcare professionals can be personalized using virtual reality and artificial intelligence tools; similarly, for example, personalized health coaching can be conducted through virtual consultations with chatbots or digital health promotion interventions can target selected audience identified through the analysis of electronic patient records.^14^

##### Automation:

Automation is intended as the use of control systems and information technologies to make a process operate automatically. Automation in healthcare management is being implemented for example with decision support systems, automated flagging of adverse clinical events,^15^ drugs dispensing systems and health services scheduling.^16^ Simpler than that, electronic health records data can largely support automatic reporting to public health agencies on notifiable diseases and chronic conditions.

##### Prediction:

Large amounts of electronic data from different sources (health data but also meteorological forecasts, social media and geographic information systems data) are used to inform both AI and non-AI prediction models that can early detect or predict, for example, adverse events, healthcare associated infections, infectious diseases’ outbreaks or health emergencies,^17–18^ allowing effective prevention and early intervention. In clinical practice, supervised and unsupervised AI-based models are increasingly used for automated image interpretation; in public health this can be applied to screening programmes.

##### Data analytics:

Digital tools brought a revolution in data gathering and sharing and in their analytics, with major applications to public health. First, the amount of available data has exponentially grown. Large volumes of structured, semi-structured and unstructured data (*big data*) are mined for information and used in machine learning projects and other advanced analytics applications, both in public health research and practice.^19^ Second, data are rapidly (*timeliness*) shared and transmitted (i.e. telemedicine or real time surveillance applications) and linked with other data sources (*interoperability*) with major potential applications to health management, public health monitoring, reporting and public health analytic research, among others.

##### Interaction:

Digital solutions allow patients, as well as the general population to generate their own health data^20^ and, most importantly from a public health point of view, to monitor and interpret them, with large impact on patients’ empowerment. Similarly, smartphone applications support behavioral risk factors (i.e. physical activity) self-management with impact on primary prevention. Internet and social media introduced interaction in communication about health (as compared with unidirectional communication of traditional media where it was not possible to share or comment on published content), this bolstering the general population’s active role in health decisions.

The features nailed down above can support advances and progress in public health practice, in public health education and training, in public health policy (both inside and outside the health sector), and in public health research. The theoretical conceptual framework we developed allows to distill the potential public health benefits and advantages brought by digitalization (Box 1), the disadvantages, risks and potential pitfalls being highlighted in other papers of the supplement.^21^^,^^22^

**Box 1 Potential Public health benefits and advantages brought by digitalization**

From a public health perspective, digitalization provides several benefits:

- It supports the transition from cure to prevention
- It helps to put people and patients at the center and supports their empowerment;
- It makes healthcare management and delivery more efficient, safer and cheaper

### Digitalization supports the transition from cure to prevention

One of the biggest gains of digital health is that it supports a shift from cure to prevention; this holding true both for primary and secondary prevention.

With regard to the former, as acknowledged by WHO, digital health technologies offer ways to self-manage health which positively impact on behavioral risk factors’ distribution. For example, a systematic review assessed the potential benefits of digital health interventions on cardiovascular disease (CVD) outcomes and risk factors and reported telemedicine, Web-based strategies, e-mail, mobile phones, mobile applications, text messaging and monitoring sensors-based interventions to reduce CVD events, hospitalizations and mortality and to lower BMI and weight, as compared with normal care.^23^ Evidence on the impact of digital preventive interventions are accumulating for communicable and non-communicable diseases including, among others, diabetes and mental health.^24–27^ With regard secondary prevention, as advances in genomics and its application to clinical routine are increasingly allowing to identify people at risk of developing diseases with genetic basis, as well as to predict response to treatment, the role of secondary prevention will become more and more central in the future. Indeed, recent EU-funded projects concluded that the potential of personalized medicine will and should step out of diagnosis and treatment and be exploited for disease prevention.^28^ As digitalization supports the system migrating from treatment toward (early) diagnosis and prevention, public health strengthens its action and role within the wider health sector.

Evidence detailing and quantifying the actual fulfillment of the theoretical potential public health benefits and advantages brought by digitalization reported in Box 1 is accumulating, but still currently lacking in many aspects. Also, besides evidence gathering, implementation of effective digital solutions in different public health domains is running at different speeds in different contexts and scale-up timing varies across settings.

To take full advantage of the potential offered by digitalization to broader public health action, minimizing its potential risks^21^^,^^22^ a whole system thinking should be put in place, encompassing all pillars described in the next section.

## Pillars for a successful European strategy for public health digitalization

The digitalization of public health should not be treated as a discrete element to focus on in silo, rather it should be considered in a broader value chain^29^ to best achieve populations’ health and wellbeing. Indeed, if—as underlined by the OECD—digital technologies are reshaping societal boundaries,^29^ a successful European strategy for public health digitalization should integrate all pillars reported in Box 2.

**Box 2 Pillars for a successful European strategy for public health digitalization**

- **Political commitment:** strong political commitment and government leadership are needed to implement digital public health strategies at the national level, and across Europe.
- **Normative and regulatory frameworks:** a clear set of regulation should support interoperability to allow safe and effective data exchange between different information and communication technology systems at the national and European level.
- **Technical infrastructure:** National and regional health authorities, as well as hospitals and healthcare agencies should be equipped with technical infrastructure to support the implementation of digital solutions.
- **Economic investments:** the successful implementation of digital public health solutions needs targeted public and private economic investments.
- **Training and education**: the public health workforce need to be trained to embrace digital solutions and university curricula should increasingly include multidisciplinary digital health modules.
- **Research**: R&D as well as operational research should be carried out drawing from health services and biomedical informatics scholarly for studies on digital health interventions' impact, efficacy and cost-effectiveness.
- **Monitoring and evaluation:** the introduction and monitoring of public health digital solutions in different settings should be supported by technology evaluations based on targeted, solid and shared Health Technology Assessment (HTA) models.

### Political commitment

The European Commission has recently published key policy documents which give clear direction to EU activities to support the digital Transformation of Health and Care for the coming years.^30^^,^^31^ The EU Communication on Digital Transformation of Health and Care in the Digital Single Market identifies three priorities: (i) Citizens' secure access to their health data, across the EU; (ii) Personalized medicine through shared European data infrastructure and (iii) Citizen empowerment with digital tools for user feedback and person-centered care. In 2018, the WHO Regional Office for Europe launched the WHO/Europe initiative for Digitalization of Health Systems as an immediate European regional action for delivery the WHA Resolution on mHealth/digital health urging Member States to prioritize the development, evaluation, implementation, scale-up and greater utilization of digital technologies, as a means of promoting equitable, affordable and universal access to health for all.^32^ At the Member States’ level, European countries are approving national eHealth and digital health policies and strategies. The latest systematic assessment carried out in 2016 reported 74% of European Member States with universal health coverage to have national-level policies or strategy on eHealth.^33^

### Normative, regulatory frameworks and technical infrastructure

Political commitment for digital public health should be translated into normative and regulatory frameworks at the European and national level. At the EU level, the eHealth Network, created under article 14 of Directive 2011/24/EU set up standards for interoperability of electronic health systems and eHealth use between Member States. At the Member States’ level, a recent study on national laws on electronic health records in EU Member States reported there are major disparities in normative frameworks across Europe.^34^ Similarly, the adequacy of available technical infrastructures to allow the operationalization of national laws varies widely. Data show that EU Member States digitize national healthcare systems at very different speeds. The distribution of a Digital Health Index that assesses countries’ digital health readiness lists Estonia and Denmark as preforming best in Europe while other countries lack far behind. Also within countries, the level of digitalization of public health services varies by region and single healthcare facility.^35^

### Economic investments

The implementation of digital solution for health requires economic investments. In 2011, the European Commission Joint Research Centre (JRC) stated that funding is essential to incentivize and promote e-health initiatives. Today, according to a recent analysis, the global digital health market accounts for $179.6 billion and is expected to grow to $536.6 billion by the end of 2025,^36^ this representing a 173.3% increase as compared to 2016. With the US and Canada still outperforming the EU, a European Commission study reported that Europe accounts for 30% of the global mHealth market, growing at the fastest pace, as compared to other regions.^37^ Key industry players in digital health are: telecommunications companies and mobile operators, big ICT and electronics groups, manufacturers of medical/monitoring devices and platforms, pharmaceutical industries and start-ups.^37^ Despite private economic interests, investments in digital health must be coupled with institutional interest and should come from regional and national health systems’ budgets, as well as the EU's so as to be sustainable and meet universal health coverage targets. Even more so in public health, the promise and potential of digital health can only be achieved if it goes hand in hand with public health objectives and it is not solely guided by market forces. Measuring public expenditure on digital public health in Europe is hampered by the heterogeneity of health systems, relying on different bodies with separate responsibilities, interests and values. Still, it is of utmost importance that national public health digital strategies are supported by adequate public funding.

### Training and education

How can the future public health workforce be prepared to act in a digitalized working environment? We expect, in general, digitalization to modify the roles and functions of staff (Supplementary appendix S2 for an imaginative representation of future public health professionals), to move the boundaries of the competencies they will be required to have. In addition, it is likely that the definition itself of public health workforce^38^ will expand to include professionals with technical background (i.e. informaticians and engineers) employed to support the implementation of digital programmes for different public health purposes. Education and training in public health should accompany these trajectories. More importantly, education and training should not give instructions on how to deal with future technologies; yet, it should aim at increasing professionals’ digital literacy,^39^^,^^40^ to make sure they will be able to embrace innovation. Although no systematic assessment has been performed, empirical data from Europe suggests elements of digital health are still rarely included in public health graduate and post-graduate programmes, the most advanced examples being in the UK. However, curricula are rapidly evolving, medical schools are including digital health credits both in clinical and non-clinical modules and selected universities offer double degrees in Medicine and Engineering.^41^

### Research

Digitalization in public health should not be an empty slogan, rather its planning, introduction and implementation should be informed by evidence. Despite strengthened political commitment and increasing economic investments in digital health technology, research outputs on its application in public health are ‘still in their infancy’. The theoretical benefits and advantages of digital public health solutions outlined in the sections above are far from being quantitatively assessed in different settings. Still, there is interest in building and accumulating evidence on the topic as demonstrated by the Horizon 2020 Work Programme 2018–2020 call on ‘Digital transformation in Health and Care’^42^ which includes 21 topics, all of which with a projected public health impact, and the recent launch of new dedicated peer reviewed journals.^43^ Research on public digital health is hampered by a number of design issues including the choice of appropriate control groups, the definition of relevant clinical, organizational and process outcomes, the transferability of its findings to different settings, without forgetting the need for being transparent and independent. Operational research should be carried out drawing from health services and biomedical informatics scholarly for studies on digital health interventions’ efficacy, effectiveness and cost-effectiveness.^44^

### Monitoring and evaluation

The impact of digital solutions on health and healthcare, once applied, must be measured and assessed. This is essential to inform decision making and resources allocation. In 2019, the European Commission Expert Panel on effective ways of investing in Health (EXPH) issued a report providing guidance on how to technically assess the impact of digital transformation of health services.^6^ In more detail, the Expert Panel reinforced that at the central and local level the decisions to adopt, use or reimburse digital health services should be based on data regarding their performance in the light of health system goals. EXPH outlined available frameworks and systematic methods for measuring the impact of the digital transformation of healthcare regarding different dimensions, including access to care, clinical and organizational outcomes, patient participation, use of resources and sustainability. In this context and with the aim of possibly establishing a European repository for evaluation methods and reports, it is important to consider how to best adapt Heath Technology Assessment (HTA) models to assess digital projects. In addition to HTA of digital solutions prior to their introduction, implementation monitoring should be carried out, reporting on how health and health systems evolve, also as a consequence of digitalization.^6^

## The role of EUPHA in leading the change towards the digitalization of public health

EUPHA, founded in 1992, is a strong, independent and broad science-based public health network, currently counting 86 members from 47 countries of the WHO European Region. As umbrella organization for public health associations, with a unique leading position in Europe, its mission is to facilitate and activate a strong voice of the public health community by enhancing visibility of the evidence and strengthening the capacity of public health professionals.^38^ Within this mission, supporting the process of effective public health digitalization has been identified within the priority areas of interest. In particular, EUPHA is committed to work to ensure that the digital potential is ‘used’ to pursue and fulfill European public health goals of improved health and well-being and narrowed health inequalities. EUPHA acknowledges the potential of digitalization as an asset *for* public health, a *means* to better deliver care and prevention and will work both to promote within its broader community the culture of ‘public health digitalization’, as well as to enable its planning, implementation and evaluation at the research, practice and policy level. On this premise in February 2019 in the context of the first ever WHO Symposium on the Future of Digital Health Systems in the European Region, EUPHA co-hosted a session on digitalization and public health, defined as the ‘Beautiful Marriage’. In the session, the extent to which digital health and public health communities are working together to co-create a healthy and fair future was discussed, concluding that the huge potential offered by joining forces is still far from being exploited. Strengthening efforts towards its fruitful exploitation, EUPHA action across Europe adds value to the ongoing efforts to achieve effective, sustainable, accessible, safe and fair public health digitalization carried out by stakeholders in regions and states, in national and international organizations, as well as by single health professionals.

Part of EUPHA action on digital public health is carried out in the context of the European Public Health (EPH) Conference (Box 3) and within EUPHA's thematic sections.

**Box 3 European Public Health (EPH) Conferences’ content on Digital Public Health**

Digital public health research policy and practice are increasingly discussed within EPH Conferences, the premier European gathering of public health professionals worldwide. During the 11th EPH Conference in 2018, which brought together over 1,675 delegates from over 72 countries from Europe and beyond, one of five plenary sessions focused on the impact of digitalization on youth health. In 2018, for the first time in EPH Conferences the topic of ‘e-health technology and communication’ was allocated a dedicated ‘track’ with 3 dedicated workshops, 2 round tables, 1 skills building seminar and 25 oral and pitch communications. In particular, one workshop featured high-level representatives of the World Health Organization and the European Commission who presented the role and action of intergovernmental organizations in supporting and guiding the digital transformation of health in Europe, together with the presentations of three selected Member States’ examples (Portugal, Estonia and Italy)^45–47^ and a representative from the next generation of public health leaders. The second workshop, chaired by EUPHA President Natasha Azzopardi Muscat and Harvard public Health professor Rifat Atun focused on the impact of technology and AI in the future of health. The 12th EPH in 2019 features two full tracks on Digital Health, developed in collaboration with the International Digital Public Health (DPH) Conference, a world leading annual interdisciplinary event bringing together experts and audiences from public health, computer and data science, MedTech industry and NGOs.

Acknowledging the importance of the current and future role of digitalization in public health and building on the enriching discussion stimulated by the EPH scientific sessions reported in Box 3, the establishment of a EUPHA thematic Section on Digital Health has been proposed with the aim of bringing together researchers, policymakers and practitioners across Europe working on digital public health for knowledge sharing and capacity building. The Section will work across competences, disciplines and settings to apply EUPHA's strategic objectives^48^ to the field of digital health. The Section will aim at building advocacy, and at gathering, producing and disseminating evidence on the need for and on the impact of digital solutions for public health. It will bring together a core group of experts in selected digital health aspects, facilitate the exchange of national-level best practices and stimulate the Europe-level debate to place digital public health high in the EU policy, research and education agenda The details of the application of EUPHA Strategy^48^ to the work of the Section on Digital Health, by single domain, is reported in table 2.

**Table 2:** EUPHA’s strategic objectives applied to the field of digital public health

Objective 1—To be a leading scientific and independent voice in the field of public health and health services research and policy	EUPHA Digital Public Health (DPH) action
**1.1. Supporting members to provide and further develop a strong national voice** ** **1.1.A. Build the capacity in advocacy ** **1.1.B. Strengthen the evidence base ** **1.1.C. Support and develop national voices ** **1.1.D. Make national voices visible at European level **1.2. Being actively involved in setting the European public health agenda** ** **1.2.A. Identify the priorities for the European public health agenda ** **1.2.B. Formulate the priorities for the European public health agenda ** **1.2.C. Voicing the priorities for the European public health agenda **1.3. Working towards global health through our work in Europe, representing the European voice at global level and supporting public health at global level** ** **1.3.A. Work towards global health ** **1.3.B. Represent the voice of Europe at global level ** **1.3.C. Support public health at global level	**Advocacy** Advocacy action for an equitable, accessible and effective application of digital technologies to public health to be developed at the national (ob. 1.1), European (ob. 1.2) and global (ob. 1.3) levels. Draw from EUPHA advocacy capacity building experience in other sectors to be applied to public health digitalization (ob. 1.1.A). Active collaboration with national Digital Health Innovation hubs, research centers and initiatives (ob. 1.1.A). Make national-level advocacy actions visible for members and countries in Europe through the DH Section webpage and other digital communication platforms (ob. 1.1.D) and during EPH Conferences. **Research and evidence gathering** Contribute to strengthen the evidence on the impact of applying digital solutions to public health: (i) facilitating research collaboration between EUPHA members and (ii) identifying research priority areas in digital public health (ob. 1.1.B). Contribute to retrieve the evidence on digital public health systematically collecting, evaluating and sistematizing upcoming relevant scientific literature on digital public health through EUPHA communication channels (ob. 1.1.B). Contribute to disseminate the evidence on digital public health through the dedicated journal sections and supplements (including European Journal of Public Health (EJPH)) (ob. 1.1.B). Develop a Support Network for Researchers within the field of Digital Health so as to ensure the further success of their work (ob. 1.1.B). **European agenda setting** Provide input and arguments to EUPHA executive bodies for inclusion of digital public health in the European Public Health agenda (ob. 1.2.A). Assist EUPHA in developing dedicated tools, partnering with other European associations with aligned values and sharing similar priorities in digital public health (ob. 1.2.B and ob. 1.2.C). Consulting and briefing EUPHA collaborating with European intergovernmental bodies (WHO and EC) currently developing European Digital Health Strategies (ob. 1.2.B and ob. 1.2.C). **Global approach** Support and participate in global initiatives on digital public health with the aim of both (i) contributing to the global debate on the topic with successful European models (ob. 1.3.B) and (ii) drawing from successful models and projects outside Europe to be adopted and adapted to European settings. Identify how digital public health tools can contribute to tackle global health threats (ob. 1.3.A and ob. 1.3.C).

**Objective 2—To build capacity and knowledge in the field of public health and health services research with the aim of supporting evidence-informed practice and policy decisions**	**EUPHA Digital Public Health (DPH) action**
**2.1. Building the capacity and knowledge of our member associations and partners, making them stronger and facilitating a growing network** ** **2.1.A. Lead by example ** **2.1.B. Support members ** **2.1.C. Help members help each other ** **2.1.D. Showcase good examples of members **2.2. Building the capacity and knowledge of the individual public health professional by further developing information exchange, joint actions and encouraging a common approach to European public health** ** **2.2.A. Support the development of strong public health and health services research at national and European level ** **2.2.B. Encourage and further develop information exchange and international capacity and knowledge building ** **2.2.C. Focus on the translation of relevant research outcomes into actual policy and practice **2.3. Building capacity and knowledge specifically in the field of practice by collaborating and further developing a system of best practice exchange**	Capacity and knowledge building • Contribute to build capacity and knowledge of members associations and individual public health professionals in the field of digital public health; encouraging the interaction between member associations' research, policy and practice (ob. 2.1, 2.2, 2.3) through: **Compiling a matrix of Expert members for different and specific areas within digital public health** (i.e. genomics and precision medicine, telemedicine, social media and health promotion) to be responsible for area-specific consulting and knowledge sharing (ob. 2.1.B and 2.1.C). **Collecting, assessing and showcasing national, regional and local actions on digital public health** (ob. 2.1.D). **Creating a European platform for: (i) operational research (ob. 2.2.A) and (ii) best practices' exchange (ob. 2.2.B) on digital public health** to inform and support practice and policy (ob. 2.2.C). Sustaining multidisciplinary collaboration **with non public health national and international partners in the field of informatics, engineering, media communication, etc. (ob. 2.2.B)**. • Developing a digitally enabled communication strategy to make **EUPHA action and outputs on digital health publicly available and useful at the national and European level** (ob. 2.2.B and 2.3). • Avoid or analytically assess and declare conflicts of interest with enterprises, companies and consulting with digital health commercial interests (ob. 2.1.A).

Objective 3 - To prepare future generations of public health professionals for their leadership role in public health	EUPHA Digital Health (DH) action
**3.1. Bringing future generations of public health professionals into the European multidisciplinary public health community** ** **3.1.A. Lead by example ** **3.1.B. Get the young active in our members **3.2. Preparing future generations of public health professionals for their leadership role, by building their capacity and knowledge** ** **3.2.A. Skills building activities ** **3.2.B. Further the network ** **3.2.C. Facilitate international exchange **3.3. Ensuring that the training and education of the new public health professional is fitting their leadership role**	**Framework for innovation leadership** Develop a guidance framework that supports EUPHA to analyze within a short time-frame the impact, benefits and harm of a new technology (ob. 3.1). Develop the right working environment and infrastructure to assist EUPHA with embracing new technologies within its workflow. This will empower EUPHA members and associations to actively follow EUPHA’s example and leadership (ob. 3.1.A). Raise the relevant funding to support the above-mentioned actions to ensure long-term sustainability. **Collaboration** Ensure the presence and active participation of EUPHAnxt representatives within the Operational Committee of the Digital Health Section (ob. 3.1.B). **Activities and outreach** Organize an annual relevant skills-building activity in collaboration with a minimum of one other Section within EUPHA. (ob. 3.2.A) Organize a EUPHA hackathon or design thinking workshop within the next 3 years to accelerate the skills and development of Digital Public Health Solutions (ob. 3.2.B) Actively attend relevant conferences within the field of Digital Health and seek to develop the relevant connections to further develop the growth of EUPHA (ob. 3.2.C) **Training and education** Collaborate with Public Health Training and Education Stakeholders (i.e. ASPHER, WFPHA, EuroNet MRPH and EUPHAnxt) to analyze the implementation of existing Digital Public Health competencies and develop an evidence-informed action plan to expand such curricula (ob. 3.3) Collaborate and raise awareness with universities and Public Health institutes about the work of the Digital Health Section (ob. 3.3)

## Funding

This research received no specific grant from any funding agency in the public, commercial, or not-for-profit sectors.

*Conflicts of interest*: None declared.

## Supplementary Material

ckz161_Supplementary_Appendix
