# Corrigendum

**DOI:** 10.1093/eurpub/ckaa083

**Published:** 2020-09-24

**Public health digitalization in Europe: EUPHA vision, action and role in digital public health** Anna Odone, Stefan Buttigieg, Walter Ricciardi, Natasha Azzopardi-Muscat, Anthony Staines

*The European Journal of Public Health*, 2019; doi:10.1093/eurpub/ckz161.

In the original article, one of author Walter Ricciardi’s affiliations (number 4) was incorrect. This has now been corrected in the online version of the article.

