# Supplementary material for: Public health digitalization in Europe: EUPHA vision, action and role in digital public health
Source: Eur J Public Health. 2019 Nov 18;29(Suppl 3):28–35. doi: 10.1093/eurpub/ckz161 (PMC6859512; doi:10.1093/eurpub/ckz161)
Supplement: ckz161_Supplementary_Appendix [file ckz161_supplementary_appendix.docx]

**Appendix A Digital Technologies for health, as classified by the Topol Review (1)**

| **Digital Health Technologies** | **Definition** |
| --- | --- |
| **DIGITAL MEDICINE** | |
| Telemedicine | Telemedicine involves the provision of clinical care from  a distance using telecommunication and information  technology, including text, audio and video consultation,  and is required to deliver the same standard of care  as face-to-face consultations |
| Sensors and wearables for remote monitoring | Sensors and wearables allow self and remote monitoring and include: point-of-care and self-tests and self-monitoring, wearables and video cameras for patient monitoring |
| Smartphone apps | Smartphone apps integrate education, symptom reporting and potential rehabilitation to improve self-management of diseases and order repeat prescriptions. They also facilitate self-monitoring illness progression and behavioral risk factors |
| Virtual and augmented reality | Virtual and augmented reality is an immersive technology combining computer-generated visual, auditory and other sensory data with the physical world. They are both tools for  the delivery of care and a platform for healthcare education |
| **AI and Robotics** | |
| Speech recognition and natural  language processing (NLP) | A branch of artificial intelligence which allows computers to understand, interpret and manipulate human written or oral language. The speech recognition is a way of dealing with the rising volume of clinical documentation; the ease and speed with which clinical documentation was completed, as well as the quality of documentation. |
| Automated image interpretation using AI | AI models which allow automated digitized image interpretation in diagnostics procedures |
| Interventional and rehabilitative robotics | Technologies which allow advanced functionality to patients with physical disability or can provide patient support |
| Predictive analytics using AI | Predictive analytics AI-based models to identify risk factors. As compared with traditional regression models, algorithms are selected automatically selected based on data. |
| **Genomics** | |
| Reading and writing the genome | “Reading the genome” is the capacity to ‘read’ an individual’s genome and capture the variation within it. It represents a unique insight into the molecular characterization of diseases. Genomic analyses will involve all areas of healthcare and impact the patient’s entire journey, from diagnosis to monitoring and treatment. “Genome-editing”, genomic engineering strategies and synthetic biology tools holds the promise of benefiting patients through the ability not only to read but also to ‘write’ genomic information. |

**Appendix B**

Future of Public Health Professional – Story

Let us imagine for a moment, in the same way as done in the Topol review (1) how the future could look like for public health professionals….

**What does the future look like for Public Health Professional?**

The Topol Review puts forward a number of case studies and even proposes how these different health professionals will advance their career. We would like to propose a similar scenario for Public Health Professionals. Let’s call this Public Health professional: Jan

**Jan in 2019 - 30 years old**

Jan is in his last year of training as a Public Health Resident at the French Public Health Institute, he has just finished off his Master of Science in Public Health and he’s currently working with two epidemiologists and a senior Public Health Specialist. On the latest Supplement of European Public Health Journal, he noted a Digital Health supplement and he was amazed and at the same time cautious about this upcoming field. He decided to explore further and see how Artificial Intelligence, specifically Machine Learning models could optimise Nutrition Policy.

**2029 - 40 years**

Jan is now a seasoned Policy Official within the Ministry of Health in Paris and there have been increasing concerns about the rise of artificial supplements allegedly replacing cooked foods across the various *arrondisements* within Paris. The Minister called in Jan and asked him about the way forward, and that he had a deadline of 4 days to prepare an augmented reality dashboard for parliament. Jan contacted his colleague in Brussel through an augmented reality set “EuroLens” and together they mapped out chloropleth maps and identified hotspots of the increased sales of this specific supplement. Jan activated the PHAI(Public Health AI tool) and asked “show me all the latest policies and legislation of meal-replacement supplements and categorise them by level of enforcement”. PHAI showcased a web of evidence highlighting in an elegant way, the shift in policy in the past 5 years and how this differed across European countries.

**2040 - 51 years**

The European Food Agency appointed Jan as their Director General after being elected through the “DirectorAI” implemented by a private company based on algorithm agreed upon by the 35 EU Member states. This AI facilitated the selection process and based the decision on Jan’s experience and the upcoming challenges of 3D printed foods. Jan requested a holograph meeting of all EU Members’ states Food Safety Directors. The different EU member states started raising concerns about defective 3D Printing Machines and nutritional content of these foods. Jan adjourned the meeting and asked his virtual assistant to set up a date for the next meeting with the Member states.

**References**

1. England HE. The Topol Review – NHS Health Education England. The Topol Review. 2019.
